# MLISB-RTK: Machine Learning Based on Inter-System Biases to Improve the Performance of RTK in Complex Environments

**DOI:** 10.3390/s26072080

**Published:** 2026-03-27

**Authors:** Ruwei Zhang, Wenhao Zhao, Xiaowei Shao, Mingzhe Li

**Affiliations:** 1 School of Aeronautics and Astronautics, Shanghai Jiao Tong University, Shanghai 200030, China; zhangrw-001@sjtu.edu.cn; 2 Beijing Research Institute of Telemetry, Beijing 100076, China; gnsslimz@foxmail.com; 3 Jiangxi Police Institute, Nanchang 330100, China

**Keywords:** RTK, ambiguity solutions, inter-system biases (ISB), challenging environments

## Abstract

In challenging environments, there often exist problems of false alarms and missed detections in real-time kinematic (RTK) ambiguity resolution, which significantly reduce the reliability and availability of position information. To address these issues, a machine-learning method is proposed to conduct a correctness check on RTK ambiguity fixing, aiming to reduce the occurrences of false alarms and missed detections. The inter-system differential RTK model is adopted. Compared with the traditional RTK model, this model can provide an effective feature, namely the differential inter-system biases (DISB), to improve the accuracy of machine-learning classification. This is because when the RTK ambiguity is correctly fixed, the DISB usually appears as a stable constant. In addition to DISB, features that are strongly related to ambiguity fixing, such as the ratio value, DOP value, and residuals, are also comprehensively utilized. This method is verified by an open-source, large-scale, and diverse GNSS/SINS dataset—SmartPNT-POS. The experimental results show that, compared with the traditional method of relying solely on the empirical ratio value for ambiguity fixing verification, the missed detection probability of this method is reduced by 2%, the false-alarm probability is decreased by 29%, and the positioning accuracy is improved by approximately 7%. Moreover, compared with other features, the DISB feature provides the highest contribution rate in the machine-learning classification model.

## 1. Introduction

RTK can provide dynamic, real-time, and high-precision positioning information. Under the premise of correct ambiguity resolution, the positioning accuracy can reach the centimeter level. However, in challenging and complex environments, the success rate of ambiguity resolution significantly decreases, and incorrect ambiguity resolution often occurs. If the ambiguity is not fixed, RTK will output a float solution positioning result, and its accuracy is mainly determined by the pseudorange accuracy, which is at the decimeter or meter level [1,2,3,4]. Incorrect ambiguity resolution is even likely to cause a positioning error of up to several tens of meters [2,3,4].

Therefore, the reliability test of the ambiguity resolution is of great significance. Excessively stringent test criteria will cause correctly fixed ambiguities to be misjudged as incorrect, which increases the false-alarm rate and thus reduces the usability of RTK positioning results. Similarly, overly lenient test criteria will allow incorrectly fixed ambiguities to pass the validation, which raises the missed detection rate and further weakens the reliability of RTK positioning results. The most commonly used verification method for ambiguity resolution is the ratio test (RT) method [1]. Due to its simplicity and effectiveness, it is widely applied in practice [4]. However, the RT method requires the use of a fixed threshold to judge the correctness of the ambiguity resolution, and this threshold is usually the empirical value of three [5]. With the development of GNSS towards multi-frequency and multi-system, the threshold of three is too conservative and likely to cause false alarms, greatly reducing the usability of RTK [5,6]. To address this, Verhagen and Teunissen proposed the fixed failure rate ratio test (FFRT) method [6,7]. This method dynamically adjusts the threshold of the RT method according to the strength of the model, balancing the reliability and usability of RTK. However, in challenging environments with multipath and non-line-of-sight (NLOS) signals, the reliability of the FFRT method significantly decreases. Hou et al. used machine-learning methods to test the ambiguity validation and achieved remarkable results [8]. However, this method requires the extraction of baseline length features, so its application is limited to static GNSS positioning. In addition, there are also methods, such as the F-test [9], W-test [10], and difference test for ambiguity testing [11]. However, these methods all assume that the ambiguity estimation is unbiased, and their performance is not ideal in practical complex environments.

Apart from the above-mentioned correctness tests based on the ambiguity domain, the ambiguity resolution can also be tested in the position domain [12,13,14]. Integrity monitoring methods mitigate the impact of biased ambiguities through geometry-based estimation and inspection and reject potentially incorrect position results [15,16,17]. However, integrity detection methods also rely on the assumption that most observations are not contaminated, and their performance is greatly reduced in challenging environments. In recent years, multi-sensor integrated navigation and positioning technology has developed rapidly and is widely used in the fields of unmanned aerial vehicles and autonomous driving [18,19,20]. The GNSS ambiguity resolution can be verified by the position results output by other sensors, but this solution requires the integration of multiple sensors, increasing the application cost.

In this paper, the ambiguity correctness test of RTK positioning results is transformed into a classification problem, with two categories: fixed solution and float solution. A machine-learning method is adopted to classify this binary classification problem. Prior to this, machine learning has been widely applied in the GNSS field and has demonstrated excellent performance [21,22]. However, most of this research focuses on GNSS space weather, with relatively few applications in positioning scenarios [23,24]. The key to the accuracy of machine-learning classification lies in the extraction of effective features [25,26]. In addition to conventional features such as ratio, DOP, and the number of satellites, this paper also extracts a key feature, differential inter-system biases (DISBs). DISBs can be calculated through the inter-system differential RTK model [27]. When the inter-system differential RTK model is used for a multi-system GNSS solution, only one unique reference satellite is selected to form the inter-system double-difference observation equation [28,29]. When DISB is estimated as a separate parameter, the model strength of the inter-system differential RTK model is exactly the same as that of the intra-system differential RTK model [30]. When the ambiguity is fixed correctly, DISB is a constant value [31,32]; however, incorrect fixing of the ambiguity will lead to large errors in the DISB results. Therefore, DISB can be used as a classification feature for the correctness test of the ambiguity. A classification method using the Random Forest algorithm is adopted to jointly utilize DISB and other GNSS features to conduct a correctness test for ambiguity resolution. The effectiveness of this method is verified through 24 sets of open-source dynamic GNSS data [33]. The experimental results show that, compared with the traditional RT method, the machine learning based on inter-system bias (MLISB-RTK) method proposed in this paper significantly reduces false alarms and missed detections, thereby improving the positioning performance of RTK. Moreover, the DISB feature provides the highest contribution degree in the classification model, exceeding the traditional ratio-value feature.

The remaining part of this paper is structured as follows: Section 2 introduces the inter-system differential RTK model and the algorithmic process of MLISB-RTK. Section 3 demonstrates the performance of MLISB-RTK in challenging environments and compares it with the RT method. Finally, conclusions are drawn in the Section 4.

## 2. Methods

### 2.1. MLISB-RTK Workflow

**Figure 1:**
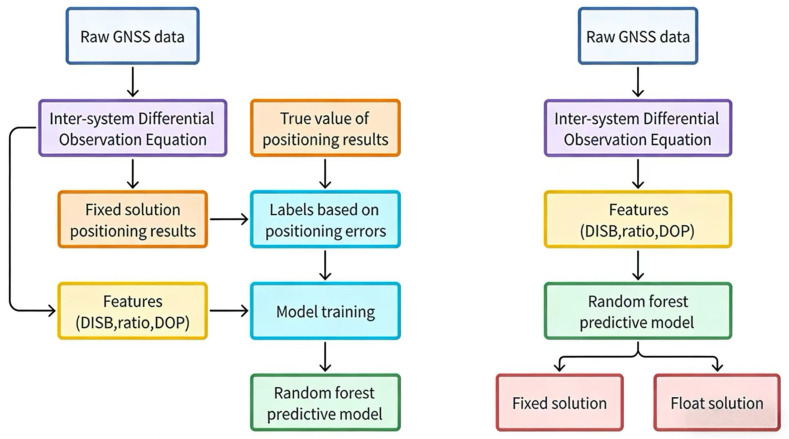
Workflow of the MLISB-RTK.

Figure 1 illustrates the comprehensive workflow of the MLISB-RTK methodology, which consists of two principal components: model training and prediction. The left panel presents the model training phase, while the right panel demonstrates the prediction implementation. The inter-system differenced RTK model is employed for positioning resolution, which provides the critical differential inter-system bias (DISB) feature for the MLISB-RTK approach, distinguishing it from conventional intra-system differenced RTK models.

During the training phase, raw GNSS observations are first processed to formulate inter-system differenced observation equations. These equations are subsequently resolved to obtain ambiguity-fixed position coordinates and DISB estimates. Notably, the Least-Squares Ambiguity Decorrelation Adjustment (LAMBDA) method is adopted for ambiguity resolution without subsequent validation of fixation correctness, directly outputting fixed solutions’ coordinates and DISB values. Concurrently, essential features, including ambiguity resolution ratio value, dilution of precision (DOP), post-fit residuals, and satellite count, are extracted during the solving process, with comprehensive feature statistics presented in Table 1.

Table 1 presents the features required for the MLISB-RTK method with their corresponding serial numbers. Feature 1 represents the number of satellites participating in the RTK resolution. Features 2–6 denote the maximum, minimum, variance, mean, and median values of the carrier-to-noise density ratio (C/N0) for all satellites, respectively. Feature 7 indicates the ratio value obtained after ambiguity fixation. Features 8–11 correspond to the geometric dilution of precision (GDOP), position dilution of precision (PDOP), horizontal dilution of precision (HDOP), and vertical dilution of precision (VDOP). Features 12 and 13 represent the proposed differential inter-system bias (DISB) characteristics in this study, specifically the differential inter-system phase bias (DISPB) and differential inter-system code bias (DISCB). Features 14–18 describe the maximum, minimum, variance, mean, and median values of satellite elevation angles. Features 19–23 characterize the maximum, minimum, variance, mean, and median values of post-fit residuals in RTK solutions. Notably, the DISPB and DISCB features represent DISB errors rather than absolute DISB values. This approach is adopted because successfully resolved DISB parameters typically demonstrate stable, constant characteristics, making their error extraction more statistically significant for analysis.

The computed fixed solutions are compared with ground truth positions acquired through commercial software post-processing. Solutions exhibiting position errors exceeding 30 cm are classified as incorrectly fixed ambiguities, while those within 30 cm tolerance are deemed correctly fixed. Each epoch’s solution is accordingly labeled as either valid or invalid ambiguity fixation based on this positional discrepancy criterion. These labels, paired with their corresponding DISC values, ratio metrics, and other discriminative features, are subsequently fed into a Random Forest classifier for model training, with optimized parameters preserved for prediction deployment.

In the prediction phase, identical feature types extracted through the inter-system differencing model are input into the pre-trained Random Forest classifier for ambiguity fixation validity determination. The system adopts a decision-conditional output strategy: ambiguity-corrected fixed solutions are directly delivered when validated, whereas float solutions are alternatively provided when potential fixation errors are detected. This dual-output mechanism ensures enhanced reliability in challenging observation environments while maintaining centimeter-level accuracy when fixation validation criteria are satisfied.

### 2.2. Inter-System Differential RTK Model

The inter-system differential RTK model serves as the core of the MLISB-RTK method. All features in Table 1 can be calculated and extracted using this model, especially the DISB feature. This section presents the mathematical derivation of the model and details the extraction methodology for DISB features.

Ignoring the atmospheric error, the single-difference observation equation of GNSS is expressed as follows [29,32]:

(1) $$\;\Delta P_{br,i}^{s_{A}}=\Delta \rho_{br}^{s_{A}}+\Delta dt_{br}+\Delta d_{br,i}^{A}+\Delta e\;\;\;$$

(2) $$\Delta \varphi_{br,i}^{s_{A}}=\Delta \rho_{br}^{s_{A}}+\Delta dt_{br}+\lambda_{i}\left(\Delta \Phi_{br,i}+\Delta \delta_{br,i}^{A}+\Delta N_{br,i}^{s_{A}}\right)+\Delta \varepsilon$$

The superscript in the formula represents satellite-related information, and the subscript represents station- and frequency-related information. Among them, b and r represent two different observation stations. $S_{A}$ represents the GNSS satellite system A. i represents the frequency corresponding to this observed value. $\lambda_{i}$ represents the wavelength corresponding to i. ΔP and Δφ represent single-difference pseudorange and phase observation values. The single differences $\Delta \rho_{br}^{s_{A}}$ represents the distance between the satellite and the receivers, $\Delta dt_{br}$ represents clock error, $\Delta d_{br,i}^{A}$ represents pseudorange hardware delay, $\Delta \Phi_{br,i}$ represents initial phase bias, $\Delta \delta_{br,i}^{A}$ represents the phase hardware delay, $\Delta N_{br,i}^{s_{A}}$ represents ambiguity, Δe and Δε represent pseudorange and phase measurement noise.

Building upon the between-receiver, single-difference observation equations, inter-satellite differencing is performed to form double-difference observation equations. Distinct from traditional intra-system, double-differencing approaches, the inter-system differential RTK model uniquely selects a reference satellite across multiple GNSS constellations. This enables the simultaneous formation of both intra-system and inter-system, double-difference observation equations:

(3) $$\Delta \nabla P_{br,i}^{1_{A}s_{A}}=\Delta \nabla \rho_{br}^{1_{A}s_{A}}+\Delta \nabla e$$

(4) $$\Delta \nabla \varphi_{br,i}^{1_{A}s_{A}}=\Delta \nabla \rho_{br}^{1_{A}s_{A}}+\lambda_{i}\Delta N_{br,i}^{s_{A}}-\lambda_{i}\Delta N_{br,i}^{1_{A}}+\Delta \nabla \varepsilon$$

(5) $$\Delta \nabla P_{br,ij}^{1_{A}s_{B}}=\Delta \nabla \rho_{br}^{1_{A}s_{B}}+\Delta d_{br,j}^{B}-\Delta d_{br,i}^{A}+\Delta \nabla e$$

(6) $$\Delta \nabla \varphi_{br,ij}^{1_{A}s_{B}}=\Delta \nabla \rho_{br}^{1_{A}s_{B}}+\left(\lambda_{j}*\Delta \Phi_{br,j}-\lambda_{i}*\Delta \Phi_{br,i}\right)+\left(\lambda_{j}*\Delta \delta_{br,j}^{B}-\lambda_{i}*\Delta \delta_{br,i}^{A}\right)\;+\lambda_{j}\Delta N_{br,j}^{s_{B}}-\lambda_{i}\Delta N_{br,i}^{1_{A}}+\Delta \nabla \varepsilon$$

Compared with the intra-system double-difference observation equations, the inter-system double-difference observation variances do not eliminate the system-related hardware delay parameters. The hardware delay parameters among them are integrated as follows:

(7) $$\Delta d_{br,j}^{B}-\Delta d_{br,i}^{A}=b_{DISCB}$$

(8) $$\left(\lambda_{j}*\Delta \Phi_{br,j}-\lambda_{i}*\Delta \Phi_{br,i}\right)+\left(\lambda_{j}*\Delta \delta_{br,j}^{B}-\lambda_{i}*\Delta \delta_{br,i}^{A}\right)=\lambda_{j}*b_{DISPB}$$

The frequencies of the observations that form the inter-system double-difference observation equations may be different, resulting in the inability to form double-difference ambiguities with integer characteristics for the ambiguities. The single-difference ambiguities need to be transformed as follows:

(9) $$\lambda_{j}\Delta N_{br,j}^{s_{B}}-\lambda_{i}\Delta N_{br,i}^{1_{A}}=\lambda_{j}\Delta N_{br,j}^{s_{B}}-\lambda_{j}\Delta N_{br,i}^{1_{A}}+\lambda_{j}\Delta N_{br,i}^{1_{A}}-\lambda_{i}\Delta N_{br,i}^{1_{A}}\;=\lambda_{j}\Delta \nabla N_{br,ij}^{1_{A}s_{B}}+\lambda_{ji}\Delta N_{br,i}^{1_{A}}$$

(10) $$\Delta \nabla N_{br,ij}^{1_{A}s_{B}}=\Delta N_{br,i}^{1_{A}}-\Delta N_{br,j}^{1_{B}}+\Delta N_{br,j}^{1_{B}}-\Delta N_{br,j}^{s_{B}}=\Delta \nabla N_{ij}^{1_{A}1_{B}}+\Delta \nabla N_{ij}^{1_{B}s_{B}}$$

In summary, the inter-system double-difference observation variances are arranged as follows:

(11) $$\Delta \nabla P_{br,ij}^{1_{A}s_{B}}=\Delta \nabla \rho_{br}^{1_{A}s_{B}}+b_{DISCB}+\Delta \nabla e$$

(12) $$\Delta \nabla \varphi_{br,ij}^{1_{A}s_{B}}-\lambda_{ji}\Delta N_{br,i}^{1_{A}}=\Delta \nabla \rho_{br}^{1_{A}s_{B}}+\lambda_{j}{\tilde{b}}_{DISPB}+\lambda_{j}\Delta \nabla N_{br,j}^{1_{B}s_{B}}+\Delta \nabla \varepsilon$$

Here ${\tilde{b}}_{DISPB}=b_{DISPB}+\Delta \nabla N_{br}^{1_{A}1_{B}}$. The DISB characteristics can be extracted using the above formula.

## 3. Experimental Validations

To assess the effectiveness of the proposed MLISB-RTK method, we conducted validation experiments using the SmartPNT-POS open-source dataset (https://www.kaggle.com/datasets/fengzhusgg/smartpnt-pos, accessed on 20 September 2025). The dataset encompasses both open-sky environments and challenging urban scenarios. In the experimental setup, we employed a GPS/BDS dual-system, single-frequency, single-epoch RTK processing mode. Consequently, dataset segments containing only GPS observations were excluded from the evaluation. Ultimately, 24 data sequences were selected for the assessment of the MLISB-RTK method, comprising a total of 20,007 GNSS epochs. To comprehensively validate the proposed method, we designed the experimental environment in two parts. In the first part, all GNSS epochs were randomly shuffled, with 80% used as the training set and the remaining 20% as the test set. In the second part, 23 out of the 24 data sequences were used as the training set, while the remaining sequence was designated as the test set. The reason for this design is that Experiment 1 can verify the effectiveness of the MLISB-RTK model on a large amount of overall data, while Experiment 2 can verify the effectiveness of the model in a single complete test task. The selected test sequence was collected in a complex environment where ambiguity resolution poses significant challenges.

### 3.1. Feature Correlation Analysis

In this section, we analyze the correlation among all features listed in Table 1 using the Pearson correlation coefficient. The Pearson correlation coefficient is widely applied in feature selection for machine-learning classification tasks as it quantifies the degree of correlation between different features. Based on this metric, dimensionality reduction can be performed to improve computational efficiency.

**Figure 2:**
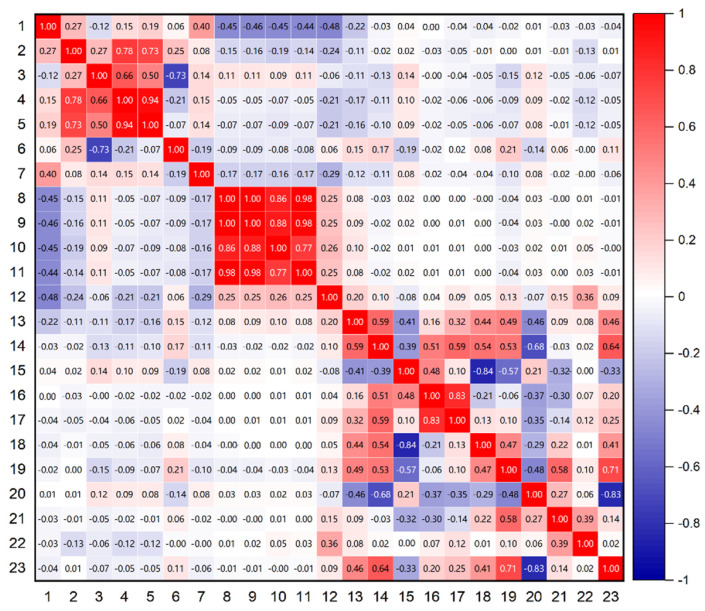
The correlations among all features.

Figure 2 illustrates the Pearson correlation coefficients for the 23 extracted features, with feature indices and names corresponding to those in Table 1. The results indicate a strong correlation among the four DOP-related features. Therefore, when deploying this algorithm on computationally constrained platforms, dimensionality reduction for these features should be prioritized. Notably, the DISPB feature maintains high independence. Additionally, the widely used ratio feature also demonstrates strong independence. Apart from correlation analysis, feature selection must also consider the contribution of each feature to classification performance. This aspect will be further explored in the next section.

Table 2 summarizes the correlation coefficients between each feature and the others, calculated from the cumulative sum of the covariance matrix coefficients in Figure 2. It can be seen from the table that the correlation coefficient of feature 12 is relatively low, and its correlation with every other feature is less than 0.5.

### 3.2. Random Split of Entire Dataset Experiment

In this section, we consolidate all epochs from 24 datasets into a single set and apply random shuffling. After shuffling, the dataset is split into training and testing sets, with 80% of the data used to train a Random Forest classification model and the remaining 20% serving as the test set for model validation [34]. The ambiguity resolution validation results of the MLISB-RTK method are compared against those of the traditional RT method.

**Figure 3:**
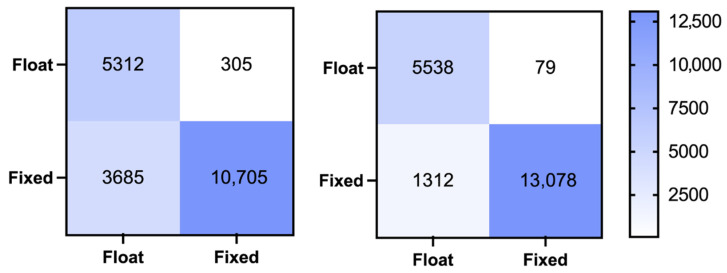
The classification confusion matrix for the random split of the entire dataset experiment by the RT method (**left**) and MLISB-RTK method (**right**).

A confusion matrix is a widely used tool in machine learning to present classification results. Figure 3 illustrates the ambiguity validation confusion matrices for both the MLISB-RTK and RT methods, while Table 3 provides detailed statistical results. The probabilities of missed detection, false detection, and overall accuracy are calculated as follows:

(13) $$R_{fd}=\frac{FN}{FN+TP}\times 100\%$$

(14) $$R_{md}=\frac{FP}{FP+TN}\times 100\%$$

$$R_{acc}=\frac{TP+TN}{TP+TN+FP+FN}\times 100\%$$

Here, $R_{fd}$, $R_{md}$ and $R_{acc}$ represent the false detection rate, the missed detection rate, and the accuracy rate, respectively. TP, TN, FP, and FN represent true positive, true negative, false positive, and false negative, respectively.

From the results in the figures and tables, it is evident that MLISB-RTK significantly outperforms the RT method in ambiguity validation. Specifically, the number of missed epochs decreases from 305 to 79, reducing the missed detection rate from 5.4% to 1.4%. The number of falsely detected epochs is reduced from 3685 to 1312, lowering the false detection rate from 25.6% to 9.1%. While the missed detection rate only improves by 4%, the false detection rate sees a much more significant improvement with MLISB-RTK. This improvement is primarily due to the conservative nature of the RT method, which tends to misclassify correctly fixed ambiguities as incorrect and, as a result, only outputs a float solution. In terms of overall classification accuracy, MLISB-RTK increases accuracy from 80.1% to 93% compared to the RT method.

**Figure 4:**
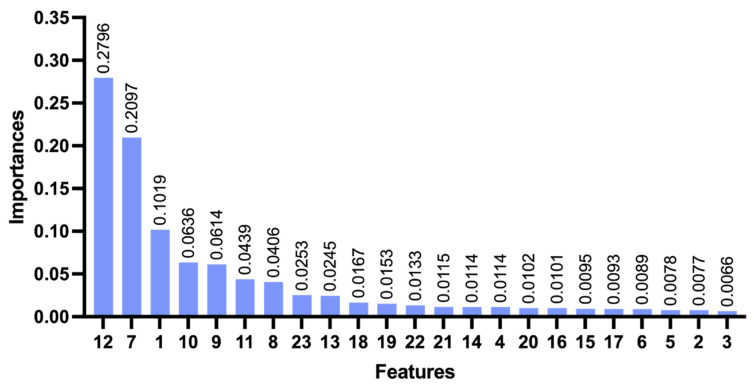
The contribution degree of all features for the random split of the entire dataset experiment.

Figure 4 presents the contribution of various features to the classification accuracy of MLISB-RTK. The classification contribution of each feature is obtained by quantifying and calculating the degree of contribution of each feature in the model after the model classification [35,36]. The results indicate that the proposed DISPB feature contributes the most, followed by the traditional ratio test value, further validating the effectiveness of the MLISB-RTK method. When deploying the MLISB-RTK algorithm on computationally constrained platforms, feature dimensionality reduction can be performed based on feature importance and correlation to optimize efficiency [37,38]. It is worth noting that feature 13 (DISCB) contributes far less than DISPB. This is because the accuracy of DISCB is determined by pseudorange accuracy at the meter level, whereas DISPB achieves centimeter-level accuracy, which better reflects classification accuracy.

### 3.3. Single-Group Split Comparative Experiment

**Figure 5:**
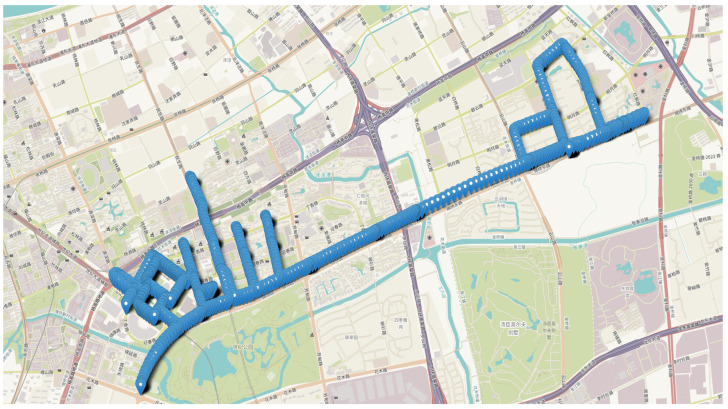
The vehicle’s running trajectory in a complex environment.

**Figure 6:**
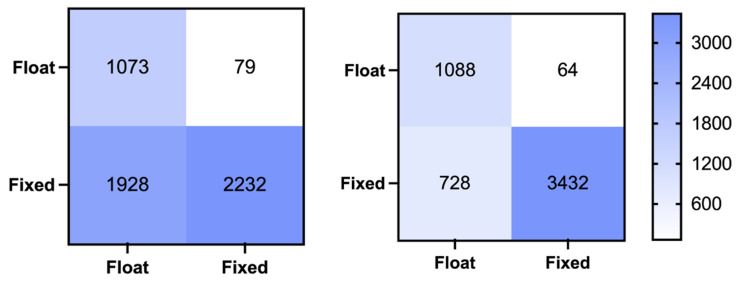
The classification confusion matrix for the single-group split comparative experiment by the RT method (**left**) and MLISB-RTK method (**right**).

In this section, we selected one particularly challenging dataset (DATA17) from the 24 available datasets for focused analysis. This dataset was collected in a complex environment, and the ambiguity fixing rate of the traditional RT method was relatively low for this dataset. The trajectory points of DATA17 from the open-source dataset are illustrated in Figure 5. We utilized the remaining 23 datasets as the training set to establish model parameters, which were subsequently applied to test this challenging dataset. Figure 6 presents the confusion matrices comparing the prediction outcomes of MLISB-RTK and conventional RT methods on this dataset, with detailed statistical results summarized in Table 4.

The experimental results demonstrate significant improvements achieved by the MLISB-RTK approach. Compared to conventional RT methods, the number of missed epochs decreased from 79 to 64 (corresponding to a reduction in missed detection probability from 6.9% to 5.6%) while false-alarm epochs were substantially reduced from 1928 to 728 (equivalent to a false-alarm probability decrease from 46.3% to 17.5%). Notably, the 28.8% reduction in false-alarm probability significantly outweighs the 1.3% improvement in missed detection rate. These findings demonstrate that the MLISB-RTK method not only effectively mitigates ambiguity resolution errors in RTK positioning but also substantially enhances solution availability.

The overall classification accuracy improved remarkably from 62.2% with conventional RT methods to 85.0% using MLISB-RTK. The improvement effects of the two experiments are comparable. The MLISB-RTK method has obvious advantages over the RT method in both cases, which also indicates that there is no model overfitting phenomenon in the experiments.

**Figure 7:**
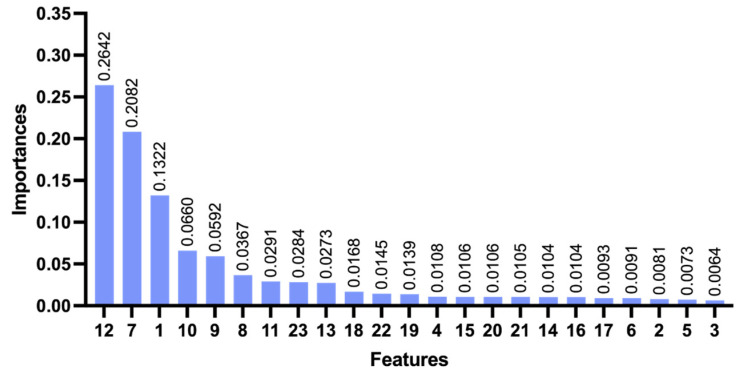
The contribution degree of all features for the single-group split comparative experiment.

Figure 7 illustrates the contribution of each feature in this dataset to the classification accuracy of the MLISB-RTK method. As observed from the figure, the DISPB feature and the ratio feature continue to provide the highest contribution. This result is consistent with those of the random split of the entire dataset experiment, further validating the effectiveness of the DISPB feature, whose advantage remains stable across different datasets.

**Figure 8:**
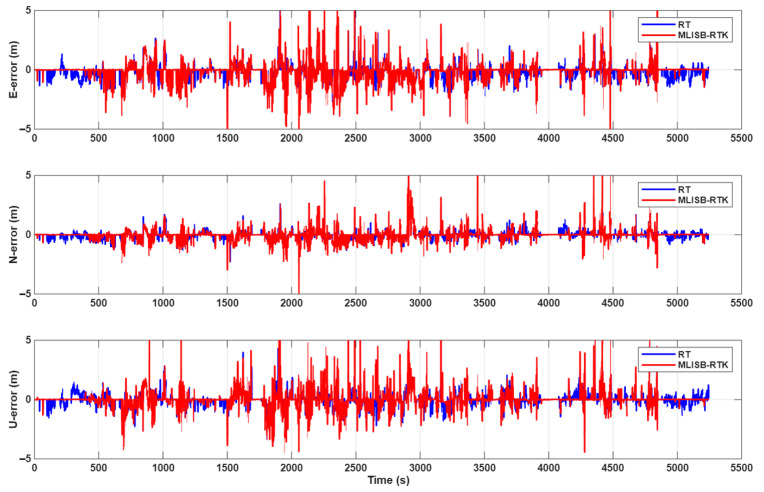
The positioning errors of the RT method and the MLISB-RTK method.

Figure 8 presents the final positioning error results obtained by the MLISB-RTK and RT methods. Both methods employ the same positioning model, differing only in the ambiguity validation approach. As shown in the figure, the positioning accuracy of the MLISB-RTK method is significantly higher than that of the RT method. This is attributed to the substantially lower misclassification probability of the MLISB-RTK method, which enables successful ambiguity resolution over more epochs. This section adopts an error analysis method that compares the positioning results with the reference coordinates. By comparing with the reference coordinates, the root-mean-square (RMS) positioning errors are calculated. The reference coordinates are obtained from GNSS/INS integrated navigation data computed by commercial software. The statistical comparison of positioning accuracy is provided in Table 5. The RMS positioning errors of the MLISB-RTK method in the E, N, and U directions are 0.88 m, 0.52 m, and 0.80 m, respectively, whereas those of the RT method are 0.94 m, 0.55 m, and 0.86 m, respectively. Compared to the RT method, the MLISB-RTK method achieves positioning accuracy improvements of 6.4%, 5.5%, and 7% in the E, N, and U directions, respectively. The improvement in positioning accuracy achieved by the proposed method is mainly reflected in two aspects: reducing the incidence of incorrect ambiguity fixing and increasing the number of correctly fixed ambiguities. The former can replace incorrectly fixed coordinates with float solutions or even correctly fixed solutions, while the latter can convert original float solutions into fixed solutions, thereby improving the overall positioning accuracy.

## 4. Conclusions

This study proposes a machine-learning-based RTK positioning method utilizing DISB features to verify the correctness of ambiguity resolution, thereby enhancing RTK positioning performance in complex scenarios. Unlike traditional RT methods for ambiguity validation, our approach employs DISB characteristics as the core feature and combines them with ambiguity-related parameters, including ratio values, satellite counts, and DOP, through machine-learning techniques. The foundation of this method lies in the inter-system differential RTK positioning model, which differs from traditional intra-system differential RTK models by providing the critical DISB feature while maintaining equivalent positioning model strength and performance outcomes.

The proposed method was validated using the SmartPNT-POS open-source dataset through two experimental designs. The first experiment (random split of the entire dataset) randomly shuffled and divided the complete dataset into training and testing sets. Results demonstrate that the MLISB-RTK method reduces false-alarm rates by 20.2%, decreases missed detection rates by 4%, and improves overall accuracy by 13% compared to conventional RT methods. The second experiment (single-group split comparative test) selected a challenging dataset collected in complex environments for testing while using other data for training. Experimental results show a 28.8% reduction in false alarms, a 1.3% decrease in missed detections, and a 22.8% improvement in overall accuracy. In both experiments, the proposed DISB feature contributed most significantly to model performance, outperforming traditional ratio features.

In conclusion, the MLISB-RTK method provides a novel approach for ambiguity validation in GNSS positioning. Compared with traditional methods, it exhibits superior performance in complex environments, especially in significantly reducing false ambiguity-fixing events. This greatly improves the availability of RTK fixed solutions and enhances positioning reliability under harsh and challenging conditions. The application of machine-learning methods offers a new perspective for the field of GNSS ambiguity validation.

## Figures and Tables

**Table 1:** All feature names and their serial numbers.

NO.	Feature	NO.	Feature	NO.	Feature
1	number of satellites	9	PDOP	17	average of EA
2	max of C/N0	10	HDOP	18	median of EA
3	min of C/N0	11	VDOP	19	max of residual
4	variance of C/N0	12	DISPB	20	min of residual
5	average of C/N0	13	DISCB	21	variance of residual
6	median of C/N0	14	max of EA	22	average of residual
7	ratio	15	min of EA	23	median of residual
8	GDOP	16	variance of EA		

**Table 2:** Correlation coefficients of all features.

NO.	Correlation	NO.	Correlation	NO.	Correlation
1	3.60	9	4.46	17	3.35
2	3.66	10	4.16	18	3.95
3	4.04	11	4.29	19	5.01
4	4.26	12	3.92	20	4.71
5	3.96	13	5.09	21	2.84
6	3.06	14	5.38	22	1.81
7	2.73	15	4.46	23	4.50
8	4.40	16	4.14		

**Table 3:** Accuracy statistics for the experiment of random splitting of the entire dataset.

Method	Miss Detection Rate	False Detection Rate	Accuracy
RT	5.4%	25.6%	80.1%
MLISB-RTK	1.4%	9.1%	93.0%

**Table 4:** Accuracy statistics for the single-group split comparative experiment.

Method	Miss Detection Rate	False Detection Rate	Accuracy
RT	6.9%	46.3%	62.2%
MLISB-RTK	5.6%	17.5%	85.0%

**Table 5:** Positioning error statistics for the single-group split comparative experiment.

Method	RMS (m)		
	E	N	U
RT	0.94	0.55	0.86
MLISB-RTK	0.88	0.52	0.80

## Data Availability

The raw data supporting the conclusions of this article will be made available by the authors on request.
